# Mathematical modelling of cross-linked polyacrylic-based hydrogels: physical properties and drug delivery

**DOI:** 10.1007/s13346-022-01129-2

**Published:** 2022-02-12

**Authors:** Filippo Bisotti, Fabio Pizzetti, Giuseppe Storti, Filippo Rossi

**Affiliations:** Department of Chemistry, Materials and Chemical Engineering “Giulio Natta”, Politecnico Di Milano, Milan, Italy

**Keywords:** Hydrogels, Drug delivery, Swelling dynamics, Mathematical modelling, Transport phenomena

## Abstract

Recently, hydrogels have gained significant importance in different applications, such as tissue engineering and drug delivery. They are 3D structures of hydrophilic polymers held together through physical or chemical crosslinking. Important is their ability to swell in presence of solvents, forming elastic gels able to maintain their original shape. Furthermore, these scaffolds slowly degrade in the physiological environment, leading the growing tissue to replace the former filled site. In this work, hydrogels have been synthetized using branched polyacrylic acid (carbomer) cross-linked with an aliphatic polyetherdiamine (elastamine). In particular, we focused on the description of their equilibrium conditions in swollen state and the dynamic simulation of the swelling process. These hydrogels exhibited a peculiar swelling behaviour characterized by an overshoot of the volume increase before reaching the equilibrium. Notably, such behaviour was found at different pH values. In this manuscript, the swelling behaviour was studied by mathematical modelling. Moreover, the ability of these devices to release drugs was also examined through a literature model to understand the different operating transport mechanisms.

**Supplementary Information:**

The online version contains supplementary material available at 10.1007/s13346-022-01129-2.

## Introduction

Hydrogels are biomedical devices that gained increasing interest as potential drug delivery carriers for advanced injury treatments [1]. These applications are possible due to their peculiar structure and physical properties [2]. Hydrogels are 3D structures made of polymer chains interconnected by a crosslinker: the backbone structure and the extent of crosslinking determine their mechanical and swelling properties [3, 4]. Indeed, hydrogels absorb a huge amount of solvent, entrapping liquid and increasing their volume even by many times [5]. If swelling is carried out in presence of a drug, the drug is released when the swollen hydrogel is put in a different medium. Hence, swelling and drug delivery are intimately interconnected [6]. Liquid absorption reaches equilibrium conditions once the tensional forces on the polymer chains are balanced by elastic forces. Therefore, mechanical properties strongly affect the swelling behaviour and resulting drug delivery performances [7].

The main parameters characterizing swollen hydrogels are mesh size, crosslinking density, average molecular weight between two following crosslinks and overall volume. The swelling kinetics describes the dynamic evolution in time of the liquid solvent uptake within the hydrogel structure [8]. Usually, the swelling dynamics is described by the evaluation of the swelling ratio ${Q}_{V}$ defined as

1 $${Q}_{V}=\frac{{W}_{s}-{W}_{d}}{{W}_{d}}$$

where ${W}_{s}$ and ${W}_{d}$ are the weights of the swollen and dry hydrogel respectively. In the literature, two possible swelling dynamics have been experimentally verified: (i) the monotonously increasing approach to equilibrium swelling, which is the most common, and (ii) the “overshoot swelling” which involves intermediate non-equilibrium configurations [9]. The latter dynamic trend is quite uncommon and exhibited by a limited set of hydrogels.

Focusing on drug delivery, as mentioned before, load-bearing capacity is one of the pivotal properties of hydrogels. In biological applications, a drug or other therapeutic molecules can be first loaded inside such structure and then released to an external medium [10]. Drug delivery systems (DDSs) are structures able to encapsulate drugs and then release them in a controlled way. In recent years, hydrogels have been used as controlled DDS, since they enable the maintenance of the drug concentration inside the therapeutic range, thus avoiding the risk of under and overdosing [11]. Not only the drug release mechanism is important for the description of the release kinetics, but also the degradation properties, since usually the overall release behaviour is a combination of diffusion mechanism and degradation (that can be bulk or by surface erosion) [12].

Moving to modelling considerations, it is not surprising that the mathematical modelling of hydrogels is attracting increasing interest since it enables their design and customization according to target applications and specifications. The physical properties of the swollen hydrogels at equilibrium, swelling dynamics and drug delivery properties can be predicted [13]. Moreover, modelling can be applied in designing hydrogel devices aimed at drug delivery, since drug released amount, releasing rate and discharging time can be also predicted. However, in both cases, such predictions rely on experimental tests, given the complex interplay between swelling dynamics and hydrogel features [14]. The complexity of the mathematical models for the swelling dynamics and drug delivery (i.e. diffusional behaviour) increases according to the task and the interest in catching specific features of the device. The most general approach should account for the simultaneous solid matrix deformation, solvent uptake and modifications in the diffusional properties of the solute. In the case of chemically bounded drugs, kinetic steps should be also considered with a further increment in the model complexity. Table 1 summarizes the state-of-art of the hydrogel modelling, organizing the different models into three main types. Detailed models (type 1) are applied to fully characterize the hydrogel properties [15, 16]: this group embodies molecular modelling [17] and describes solid matrix deformation [18, 19]. All the authors emphasize the capability of such models to accurately describe the hydrogel properties but also acknowledge the correspondingly demanding implementation and numerical solution. Moreover, the comparisons of the predictions of such models with experimental data are still limited, and wider general validation is still required. The models of type 2 are first principles and typically imply the numerical solution of partial differential equations (PDEs). It is often difficult to properly define boundary conditions, and large computational costs are invariably involved. For these reasons, commercial software packages are typically used in this case (e.g. COMSOL or ANSYS) [20–22], implementing numerical methods aimed at reducing the system dimensions (e.g. finite volumes) [22–24]. The type 1 model group collects the general models available in the literature both for the drug diffusion (de)coupled with the hydrogel swelling and matrix deformation without any further detail or constitutive laws to characterize the diffusion fluxes and the solid deformation. The molecular modelling is part of this category since it aims at determining macro-scale properties just considering fundamental interaction and a priori principles at the micro-scale. These models are the most general, and they represent the basis for the type 2 model. Instead, type 2 class includes the rigorous models where diffusion fluxes are empirically defined. The list of cited works reports also theoretical papers where PDEs numerical methods and algorithms are described. The last type (type 3) of models is actually a large set of semi-empirical relationships available to correlate experimental data [25–29]. This approach appears intrinsically weak since it is not general based on first principles. On the other hand, type 3 models ask for negligible computational effort while being quite efficient in representing experimental observations. As such, they can be considered helpful tools to quickly identify peculiar system behaviours minimizing complexity. Their major strengths are (i) excellent reliability for specific applications, those for which they were tailored; (ii) easy implementation and (iii) quantitative results enabling data analysis at no computational cost. Combined with a reasonable experimental campaign, these correlative models are well established and widely accepted in the literature. More recently, machine learning (ML) has been also exploited to apply such simplified modelling to large datasets, thus reducing the intrinsic weakness of this model type.

**Table 1:** Model classification

Model type	Advantages and disadvantages	References
1	General models, detailed description of mass transfer, swelling dynamic and solid matrix deformation. Often based on molecular modelling, thus using a priori principles and fundamental physical laws Very time consuming (they aim at predicting macroscale properties by modelling all the possible microscale interactions)	[15–19]
2	Usually less detailed than type 1 but still first principle methods, hence also general but at lower degree of complexity (assumptions and empirical laws typically applied while ensuring enough accuracy) Computationally demanding (PDE have to be numerically solved; CFD commercial codes as well as ad hoc numerical methods are applied)	[20–24]
3	Semi-empirical relationships tailored for specific cases; not general but versatile, flexible and non-time demanding; especially effective when limited experimental information is available; also useful to drive the experimental design Lack of generality	[25–30]

Following the above classification, a correlative model of type 3 is applied in this work to describe the swelling behaviour of a hydrogel. Our final aim is to develop a simple tool capable to explain the influence of relevant parameters on the hydrogel design, giving us the opportunity to tailor the final device according to specific needs.

In this work, the potential of mathematical modelling to determine physical properties of swollen hydrogels and interpret the swelling dynamics will be assessed, with emphasis on the interpretation of non-equilibrium states. Hydrogel samples made of carbomer (polyacrylic acid) as main constituent and elastamine RE-2000 as crosslinker will be examined [31]. Moreover, a stochastic method will be applied for characterizing the drug delivery of rhodamine B from the same hydrogel samples. In this last section, it will be proven that it is possible to apply simplified models and extend the proposed approach also to over-swollen hydrogels.

## Experimental part

The experimental data used in this work have been previously published by Mauri et al. [31], and the experimental setup and procedures are only briefly summarized in the following.

### Hydrogel synthesis

Carbomer has been dissolved into 1.5 mL of distilled water, while elastamine RE-2000 in 8.5 mL. Different proportions have been tested, as reported in Table 2. Using a syringe, the two solutions have been mixed, and a given volume has been inserted into steel cylinders (diameter 1.1 cm) and refrigerated at − 20 °C for 8 h. At this point, a physical gel is already formed. Before promoting the transition from physical to chemical crosslinking, the prepared hydrogels have been lyophilized. Such transition occurs after 25–30 min in a microwave oven (maximum admissible temperature of 80 °C).

**Table 2:** Formulations of all the samples studied in this work

**Sample name**	**Water [mg]**	**Carbomer [mg]**	**Elastamine [mg]**
2–2	10	140	1200
1–2	10	70	1200
1–3	10	70	1800
1–4	10	70	2400

### Swelling experiments

Swelling tests have been performed by weighting the dried chemically cross-linked gels and soaking them in a known volume of distilled water. Different pHs have been tested, controlling them using a buffer solution as aqueous medium. At fixed time points, the swollen hydrogels have been weighted, and the swelling ratios have been evaluated.

### Drug release experiments

In these tests, rhodamine B (RhB) has been chosen as drug mimetics. The drug loading has been obtained by soaking the dried gels into a solution of RhB in distilled water. After equilibrium has been reached, the supernatant has been withdrawn and analysed through UV–Vis at the excitation wavelength of RhB in water, exploiting the Lambert–Beer law [32], and the quantity of absorbed drug has been estimated. Then, the loaded hydrogels have been immersed in a given volume of water (or buffer solution, PBS for physiological conditions). Release assays have been performed by withdrawing a known volume of supernatant and replacing it with fresh distilled water, to maintain the concentration gradient. The withdrawn solution has then been analysed by UV–Vis and the final percentage of release estimated.

## Modelling part

### Equilibrium swelling

Before analysing the swelling dynamics, the swelling behaviour at equilibrium is discussed. The two key parameters for describing the hydrogel properties at equilibrium are mesh size ($\upxi$) and crosslinking density ($\upnu$). The theoretical model used has been derived from the Flory-Rehner theory [33] under the following simplifying assumptions:

- The number average molecular weight of the backbone (${M}_{n}$) is very large
- The volume fraction of polymer before swelling is equal to unity, since the sample underwent lyophilization

Accordingly, the Flory-Rehner equation becomes

2 $${M}_{c}=\frac{\frac{{\Omega }_{1}}{\overline{\nu }}\left({\Phi }_{2,s}^{^1/{_3}}-\frac{{\Phi }_{2,s}}{2}\right)}{\mathit{ln}(1-{\Phi }_{2,s})+{\Phi }_{2,s}+{\chi \Phi }_{2,s}^{2}}$$

where $\overline{\nu }$ is the specific volume of the backbone polymer, ${\Omega }_{1}$ the molar volume of the swelling medium, ${\Phi }_{2,s}$ the polymer volume fraction after swelling, $\chi$ the Flory polymer–solvent interaction factor and ${M}_{c}$ the average molecular weight between crosslinks.

The mesh size $\xi$ can then be evaluated given the end-to-end distance of the solvent-free state ${r}_{0}$, in turn estimated by Eq. (3) [34, 35]:

3 $${r}_{0}= {C}_{n}^{^1/{_2}}l{\left(\frac{{2M}_{c}}{{M}_{r}}\right)}^{^1/{_2}}$$

where ${C}_{n}$ is the characteristic Flory ratio (available in the literature for many polymers) and $l$ the characteristic monomer length of the bond along the polymer backbone (i.e. C–C bond). Once this distance is known, $\xi$ and $\nu$ can be evaluated through Eqs. (4) and (5) [34, 36], respectively:

4 $$\xi ={r}_{0}{\Phi }_{2,s}^{^1/{_3}}$$

5 $$\nu =\frac{{\rho }_{p}}{{M}_{c}}$$

### Swelling dynamics

Carbomer shows a completely different swelling dynamics with respect to most of the hydrogels previously analysed in the literature. Namely, instead of the classical sigmoidal curve, an overshoot is observed before reaching the equilibrium condition. This can be referred to as “overshooting effect”. The actual presence and the intensity of such overshoot are a function of different parameters, such as crosslinking degree, temperature, ionic strength and pH of the solution, and the release of unreacted polymer during swelling. The model used in this work to describe the swelling dynamics has been developed in previous works by Diez-Peña et al. [21, 22]. According to NMR experimental results, they proposed a model able to consider different “types” of water with different mobilities depending on the soaking pH medium. The same authors applied this model to N-iPPAm-co-MAA copolymers, which exhibited overshooting effect during the swelling process. Given the similar functional groups (i.e. similar interactions between the functionalities and the surrounding medium) on the backbone chains and the similar swelling behaviour, the same approach has been adopted here for carbomer-based hydrogels. According to the model, water molecules are classified into different classes, and a kinetic scheme has been proposed to describe the transformation of the different kinds of water within the system. The water molecules classification is slightly modified to fit our system:

1. Initially, the functional groups (carboxylic moieties for the carbomer hydrogel) are unbounded leading to a non-stiff structure enabling water uptake by instantaneous diffusion inside the system. Water absorption starts, and the overall water amount that the hydrogel is able to store is denoted as *A*_1_.
2. Water molecules continuously diffuse inside the gel causing its volume growth. In this phase, the water is continuously absorbed and distributed within the cross-linked structure. This type of water is referred as *A*_2_, and the corresponding hydrogel structure is named “primary structure”. It should be noted that *A*_2_ is a “product” of the water initially uptaken (*A*_1_), while its initial amount is negligible.
3. While swelling goes on, the medium pH plays a significant role since it promotes the rearrangement of the hydrogel structure through hydrogen bonding and/or electrostatic interactions. Indeed, the carboxylic moieties suitably appear in the either protonated or deprotonated form according to the medium pH. The resulting internal interactions lead to a more compact and rigid structure called “secondary structure” and the successive shrinkage. The structure stiffening is responsible for the overswelling response: the entrapped water inside the over-swollen hydrogel is named *A*_3_.
4. Finally, the stiffening process leads to structure shrinkage since the water content is higher than the equilibrium value. Part of the previously entrapped water (*A*_3_) is released, and this quantity will be referred to as *A*_4_.

Given this mechanistic picture, the following kinetic scheme involving the “transformation” of water types has been proposed:

$$A_1\overset{k_1}{\underset{k_2}\rightleftarrows}A_2\overset{k_3}{\underset{k_4}\rightleftarrows}A_3\overset{k_5}{\underset{k_6}\rightleftarrows}A_4$$

Six different kinetic constants are involved, one for each step in the kinetic scheme. However, as highlighted in the previous cited works, since the initial water uptake is driven by fast water absorption and diffusion, the reverse reactions (water release and water back diffusion) are negligible. Hence, the first two kinetic steps can be considered as irreversible. The scheme can be simplified as follows, with a single reversible step:

$$A_1\xrightarrow{k_1}A_2\xrightarrow{k_3}A_3\overset{k_5}{\underset{k_6}\rightleftarrows}A_4$$

The dynamics of the different types of water has been described through the following set of differential equations:

6 $$\frac{d{A}_{1}}{dt}=-{k}_{1}{A}_{1}$$

7 $$\frac{d{A}_{2}}{dt}={k}_{1}{A}_{1}-{k}_{3}{A}_{2}$$

8 $$\frac{d{A}_{3}}{dt}={k}_{3}{A}_{2}-{k}_{5}{A}_{3}+{k}_{6}{A}_{4}$$

9 $$\frac{d{A}_{4}}{dt}={k}_{5}{A}_{3}-{k}_{6}{A}_{4}$$

where ${k}_{1}$ is the rate constant of the transformation of ${A}_{1}$ into ${A}_{2}$, ${k}_{3}$ the one of the second irreversible reaction (over-swelling state), and ${\mathrm{k}}_{5}$ and ${\mathrm{k}}_{6}$ are those of water uptake and excess water release, respectively. The swelling ratio evaluated on a mass basis is defined as:

10 $${Q}_{t}=\frac{{m}_{t}-{m}_{0}}{{m}_{0}}=\frac{{m}_{w}}{{m}_{0}}$$

where ${m}_{0}$ is the initial mass, ${m}_{t}$ the mass at time *t* and ${m}_{w}$ the total amount of water entrapped inside the hydrogel at the same time. The last quantity is the sum of the water in the primary and secondary structure, that is ${A}_{2}$ and ${A}_{3}$. As soon as the swelling equilibrium is established, the swelling ratio is equal to the value of ${A}_{3}$. Taking advantage of the analytical solution of the previous set of differential equations, the following expression is worked out:

11 $$\begin{aligned}{Q}_{t}=\bigg[&\frac{{k}_{3}}{{k}_{3}-{k}_{1}}\frac{{k}_{1}-{k}_{6}}{{k}_{1}-{k}_{5}-{k}_{6}}{e}^{-{k}_{1}t}+\frac{{k}_{1}}{{k}_{1}-{k}_{3}}\left({e}^{-{k}_{3}t}-{e}^{-{k}_{1}t}\right)\\&-\frac{{k}_{1}}{{k}_{1}-{k}_{3}}\frac{{k}_{3}-{k}_{6}}{{k}_{3}-{k}_{5}-{k}_{6}}{e}^{-{k}_{3}t}\\&+\frac{{k}_{1}}{{k}_{1}-{k}_{5}-{k}_{6}}\frac{{k}_{5}}{{k}_{5}+{k}_{6}}\frac{{k}_{3}}{{k}_{3}-{k}_{5}-{k}_{6}}{e}^{-\left({k}_{5}+{k}_{6}\right)t}\\&+\frac{{k}_{6}}{{k}_{6}+{k}_{5}}\bigg]{a}_{0}\end{aligned}$$

where ${a}_{0}$ is the initial amount of water inside the hydrogel. At equilibrium (very long times), the last equation reduces to:

12 $${Q}_{t}(t=\infty )={Q}_{eq}=\frac{{k}_{6}}{{k}_{6}+{k}_{5}}{a}_{0}$$

Equations (11) and (12) involve five adjustable parameters: the four kinetic constants and the initial water content.

### Drug delivery

Two different regimes have been taken into consideration when dealing with drug release, the operative regime and the so-called anomalous one.

As far as the operative regime is concerned, the drug diffusion coefficient can be evaluated from the Stokes–Einstein equation [38]. The effective diffusivity inside the hydrogel matrix D_g_ can thus be evaluated using a correlation proposed by Lustig and Peppas [39]:

13 $$\frac{{\mathcal{D}}_{g}}{{\mathcal{D}}_{0}}= \left(1-\frac{{r}_{s}}{\xi }\right)\mathrm{exp}\left(-Y\frac{\Phi }{1-\Phi }\right)$$

where ${\mathcal{D}}_{0}$ is the diffusion coefficient at infinite dilution (i.e. the one evaluated with the Stokes–Einstein equation), ${r}_{s}$ the hydrodynamic diameter of the solute, $\xi$ the hydrogel mesh size, $\Phi$ the polymer volumetric fraction in the swollen hydrogel and $Y$ the ratio of the critical volume required for a successful translational movement of the solute molecule and the average free volume per molecule of liquid. According to Lin et al. [40], $Y$ can be assumed equal to unity without loss of accuracy. Once the diffusion coefficient has been evaluated, and given relaxation time and characteristic length, it is possible to evaluate the Deborah number ($\mathrm{De}$), defined as the ratio between relaxation and diffusion characteristic times [40–42]. If Deborah number is smaller than 1, the rate determining step is diffusion, while the drug release is governed by the swelling process when Deborah number is larger than 1.

Moving to the anomalous diffusion regime, different models have been proposed in the literature [40, 42, 43]. For instance, a general approach has been developed by Korsmeyer and Peppas [29, 44, 45] using the following power law fitting function:

14 $$\frac{m(t)}{m_{eq}}=k{\cdot t}^n$$

where $m\left(t\right)$ is the mass of drug released at a specific time, and ${m}_{eq}$ is the total released mass when equilibrium conditions are reached. Despite its simplicity, this approach is effective to predict the initial drug release profile (i.e., until *m*(*t*)/*m*_*eq*_ < 0.60). On the other hand, it fails systematically to describe experimental observations when drug delivery is approaching equilibrium conditions, as it will be verified in the next section. This limitation may be explained considering the lack of physical meaning of the adjustable parameters appearing in its formulation.

For different hydrogel geometries, Liu and Metters [25] demonstrated the existence of lower and upper limits of exponent *n* and associated such threshold values to the corresponding drug release regime (i.e., diffusion- or swelling-controlled release process; see Table 3). For *n* values falling within these boundaries, the so-called “abnormal” drug diffusion takes place, corresponding to a combination of the two limiting behaviours, diffusion- and swelling-controlled. In the present work, the hydrogel samples are reassembled as cylindrical elements.

**Table 3:** Values of exponent *n* and drug release regime for different geometries according to Eq. (14)

**Solid matrix geometry**	**Diffusion-controlled**	**Swelling-controlled**
Slab	0.50	1.00
Cylinder	0.45	0.89
Sphere	0.43	0.85

In this work, the model proposed by Kosmidis et al. [46–48] has been selected. Such model is based on the Weibull function:

15 $$\frac{m(t)}{{m}_{eq}}= 1-exp\left(-a{t}^{b}\right)$$

As emphasized by the same authors, a clear physical meaning has been assigned to each parameter appearing in Eq. (15) by Monte Carlo simulations. Parameter *a* is directly related to the probability of the drug molecule to escape from the solid matrix, whereas the geometrical coefficient *b* reflects the specific surface per unit of volume (m^2^/m^3^). As expected, the larger is the specific surface, the higher is the probability of drug escape from the hydrogel. Differently from the power-law approach, this method enables to properly represent the profile of the entire release curve, as will be proven in the next section.

## Results and discussion

### Equilibrium swelling

The numerical values of the main parameters estimated for the different hydrogels are summarized in Table 4.

**Table 4:** Parameter values for swelling equilibrium estimated from experiments

**Samples**	**Equilibrium swelling** *𝐐*_*𝐯*_ ( −)	**Polymer volume fraction** *ϕ* ( −)	**Mesh size** *ξ* (𝐧𝐦)	**Molecular weight** *𝐌*_*𝐜*_ (𝐠 𝐦𝐨𝐥^−1^)	**Crosslinking density** *ν* (μ𝐦𝐨𝐥 𝐜𝐦^−3^)
pH = 3					
2–2	7.20	0.139	13.66	11,348	123.37
1–2	7.63	0.131	15.15	13,416	104.35
1–3	7.92	0.126	16.15	14,896	93.98
1–4	7.32	0.137	14.07	11,910	117.50
pH = 7					
2–2	13.69	0.073	42.43	70,200	20.07
1–2	11.28	0.089	30.07	40,750	34.36
1–3	10.29	0.098	25.63	31,620	45.38
1–4	8.74	0.114	19.25	19,800	70.72
pH = 10					
2–2	8.74	0.114	19.25	19,800	70.71
1–2	10.53	0.095	21.20	23,155	60.46
1–3	7.96	0.126	16.31	15,140	92.49
1–4	6.13	0.163	10.25	7110	196.95

To check the consistency of the estimated swelling parameters, these results have been compared to those reported in the literature as evaluated by the same equilibrium model for a similar carbomer [49]. All the parameter values exhibit the same order of magnitude, and this finding supports the reliability of the applied approach.

Focusing on crosslinking density, largely different values are estimated for the same sample depending on the used external medium. In particular, at neutral pH, each hydrogel is weaker and less compact than at higher or lower pH, with variations up to one order of magnitude. Since the chemical crosslinking is constant for all carbomer samples, an additional “force” is operating able to intensify the interactions among the polymer chains and making the structure stiffer. This increase of stiffness is reflected by an increase of “effective” crosslinking density, where the term effective is used to remember it accounts for both chemical and physical crosslinking. The physical crosslinking is the key of this phenomenon: its effect overlaps the one of the chemical crosslinking or, even better, its contribution prevails forming strong physical interconnections among the polymer chains that effectively support and toughen the matrix. This consideration will become clearer when discussing the experimental results of swelling dynamics.

As a final remark, some authors (e.g. [34]), taking advantage of the theoretical framework previously developed by Schaefer [50], have investigated the relation between mesh size and polymer fraction. The simplest proposed model is the power-law relationship reported:

16 $$\xi=\alpha+\beta\cdot\phi^n$$

**Fig. 1:**
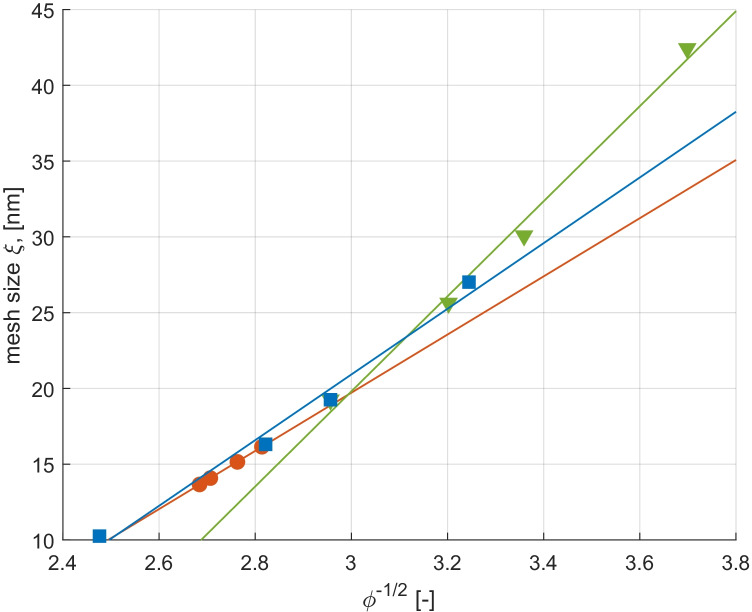
Fitting curves (solid lines) and estimated mesh sizes (dots) for the different carbomer hydrogels. Vertical dashed line stands for the upper limit in polymer volume fraction as suggested in [44]

Despite its simplicity, this power-law works properly in predicting the evolution of the mesh size with the polymer volume fraction for $n=-0.50$ and $\upphi <0.10$ regardless of the solvent and polymer nature. Our results further support this capability and indicate that the validity range of the model can be slightly enlarged, as well proven by the results in Fig. 1 and Table 5. Note that data in this figure are those previously presented in Table 4.

**Table 5:** Parameters in mesh size fitting

**pH = 3**			**pH = 7**			**pH = 10**		
*α*	*β*	*𝐑*^2^	*α*	*β*	*𝐑*^2^	*α*	*β*	*𝐑*^2^
− 37.87	19.20	0.999	− 74.93	31.51	0.994	− 44.10	21.67	0.985

### Swelling dynamics

**Fig. 2:**
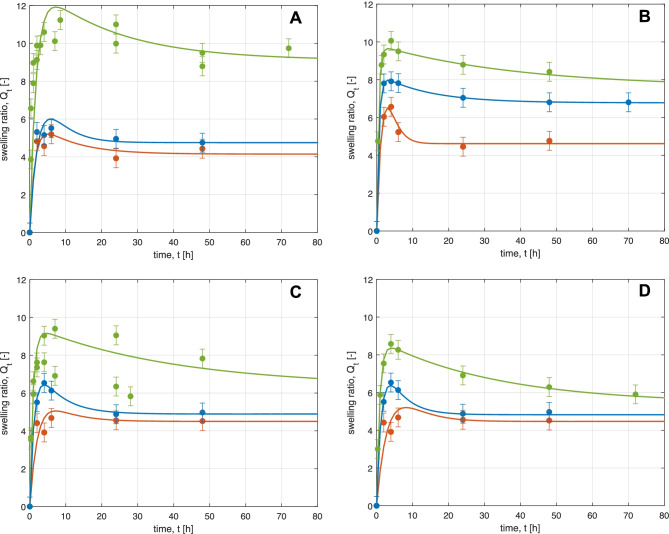
Effect of pH on swelling dynamics of all the samples: carbomer 2–2 **A**, 1–2 **B**, 1–3 **C** and 1–4 **D**. Curves are model results, symbols experimental data. Different colours identify the pH values: red = 3, green = 7, blue = 10

Experimental data have been taken from the work of Mauri et al. [31]. The comparison between model predictions and experimental results is shown in Fig. 2. Note that even though the overall experimental time was 350 h, the time scale in the figure is limited to 80 h in order to better visualize the overshoots. The five adjustable parameters of the swelling model have been estimated by fitting the experimental data at different conditions, namely different carbomer to elastamine ratios and different pH values. The latter values have been set from basic (pH = 3) to neutral (pH = 7) and to acidic conditions (pH = 10) using a buffer solution to maintain constant the solution pH to the specific value all along each experiment. The specific concentrations, along with the names of the different samples, have already been reported in Table 2. The experimental data are well described by the calculated curves whose refitted parameters are collected in Table 6. The refitted values are similar to those proposed in the literature [37, 51]; in particular, the order of magnitude of each parameter and the corresponding ratios are preserved. The largest discrepancy compared to the literature results is present in the parameters *k*_1_ and *k*_3_. On the other hand, the specific hydrogel formulation (polymer to crosslinker ratio) and the different buffer solution pH adopted in the several experimental campaigns may justify this difference. Indeed, in the light of the effect of the solvent in the interaction among polymer chains and its impact on the final hydrogel features, it is possible to conclude that these parameters may deeply affect the initial kinetic steps (water absorption and water distribution rate identified with *k*_1_ and *k*_3_ respectively) where the contact between solvent molecules and backbone structure is unavoidable.

**Table 6:** Numerical values of the fitting parameters at different pH medium for carbomer samples

**Sample**	*𝐤*_1_ (𝐡^−1^)	*𝐤*_3_ (𝐡^−1^)	*𝐤*_5_ (𝐡^−1^)	*𝐤*_6_ (𝐡^−1^)	*𝐚*_0_ (𝐦𝐋)	*𝐑*^2^	***t***_**peak**_** (h)**
pH = 3							
2–2	0.71	0.10	0.50	1.25	5.81	0.953	5.00
1–2	1.03	0.23	0.52	0.81	7.45	0.985	3.20
1–3	0.45	0.15	0.50	1.51	6.00	0.904	8.00
1–4	0.27	0.20	1.50	1.91	8.00	0.850	7.00
pH = 7							
2–2	0.55	0.045	15.0	35.0	13.0	0.985	7.00
1–2	0.95	0.080	19.1	51.3	10.5	0.925	6.00
1–3	1.10	0.025	25.0	50.0	9.50	0.874	3.50
1–4	1.15	0.030	35.7	58.0	8.75	0.996	3.50
pH = 10							
2–2	0.40	0.20	1.50	1.90	8.50	0.933	5.50
1–2	0.80	0.12	0.62	1.90	8.95	0.984	4.50
1–3	0.80	0.15	0.80	1.50	7.50	0.997	4.25
1–4	0.72	0.20	1.50	2.28	8.00	0.997	4.25

Furthermore, by analysing the experimental and model results in Fig. 2, it can be noticed that:

- In acidic and basic environment, the hydrogel structure exhibits a sort of intrinsic stiffness, related to the formation of hydrogen bonds at low pH and of electrostatic repulsive forces at high pH. From the experimental data, it seems that the hydrogen bonds stabilize the structure more effectively than the electrostatic interactions, even though both chemical and physical crosslinks cooperate to hydrogel shrinking. Structure stiffening is possible thanks to either the protonated form of the carboxylic groups, responsible for hydrogen bonds at low to medium pH or the deprotonated form, which actives electrostatic forces.
- At neutral pH, both types of physical crosslinking are weak; hence, swelling dynamics is more pronounced. At these conditions, both hydrogen bonds and electrostatic forces are operative, but at much smaller extent compared to the previous cases. As soon as the overshoot is reached, the elastic contribution of swelling starts to play the dominant role, shrinking the structure and reducing the achievable equilibrium value. In fact, such value is larger than the one established at low or high pH.
- Crosslinking density plays a key role in determining the swelling equilibrium, and its effect is more evident at pH near to neutrality. Looking at the time evolution of ${Q}_{t}$, the higher the crosslinking density, the lower the swelling, both in terms of peak width and equilibrium plateau. Such effects become less relevant in acid and basic conditions, where physical entanglement exceeds the chemical ones.
- Increasing the chemical crosslinking, the impact of the physical one on swelling equilibrium decreases.

A sensitivity analysis enables to deduce the impact of the different model parameters:

- ${k}_{1}$ controls the swelling-time derivative at the origin; furthermore, it affects the curvature near the maximum. This is consistent with the reported kinetic scheme, since the first reaction involves the initial uptake of water. The larger is the initial water uptake, the higher is the swelling rate (i.e. the initial slope).
- The ratio between ${k}_{1}$ and ${k}_{3}$ influences the width of the overshoot, while the value of ${k}_{3}$ determines the peak position. Again, this is consistent with the model, since the second step in the kinetic scheme accounts for the diffusion of the solvent into the solid matrix. In case of slow rate, the overswelling peak is reached at longer time.
- ${k}_{5}$ and ${k}_{6}$ influence the height of the final plateau, as expected according to the previous considerations on swelling equilibrium.
- ${a}_{0}$ regulates the width of the peak with respect to unswollen conditions, since the initial water content affects swelling dynamics and overshoot extent.

**Fig. 3:**
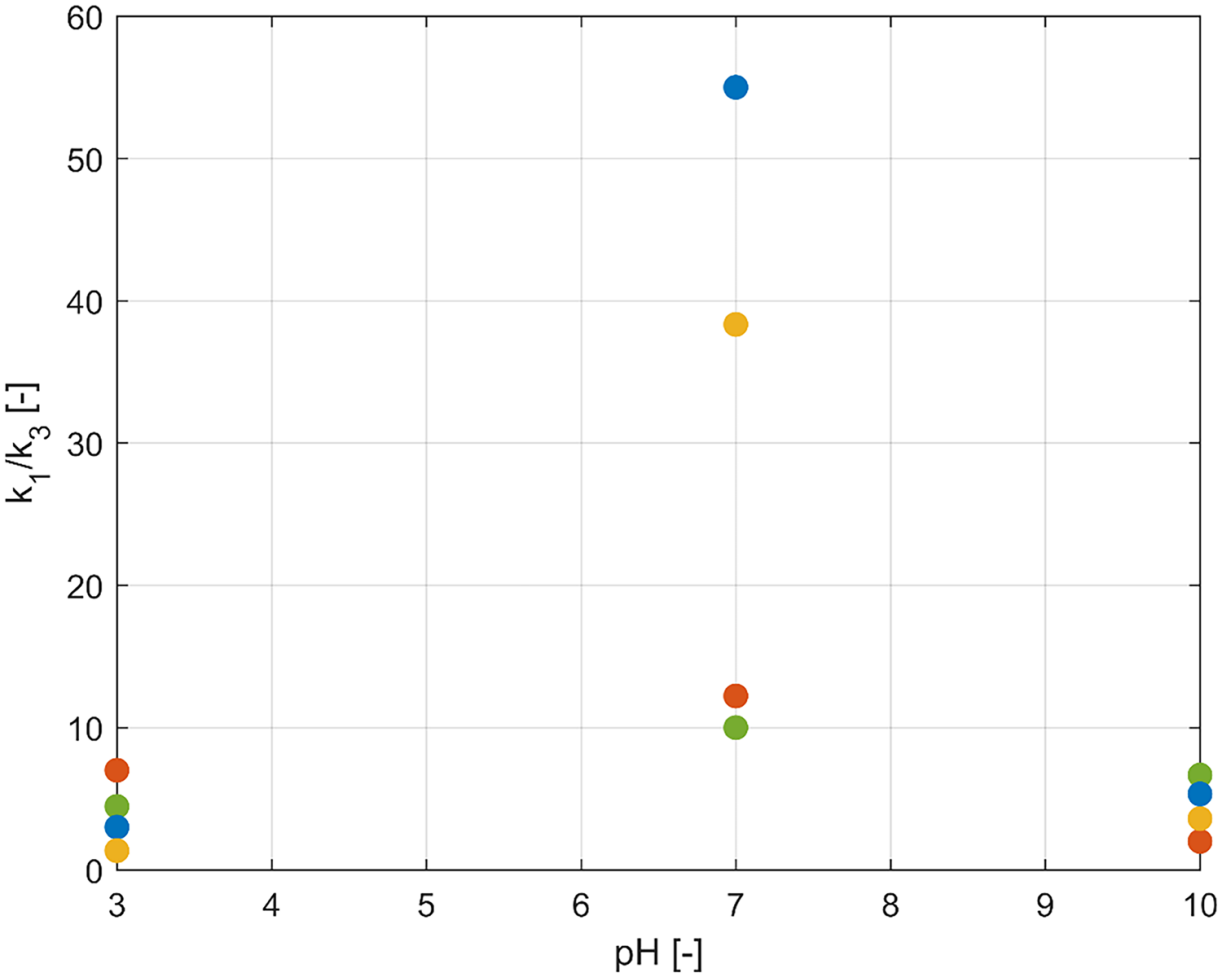
Effect of pH on the ${k}_{1}/{k}_{3}$ ratio for each sample: 2–2 (red), 1–2 (green), 1–3 (blue), 1–4 (yellow)

Figure 3 shows how the ratio between the first two rate parameters is function of pH and of the formulation used in the hydrogel production. The crosslinking density plays a relevant role at pH close to neutrality, while the differences between the different formulations are negligible at acidic and basic pH. It is important to remind the physical meaning of these two parameters: while ${k}_{1}$ determines the initial slope of the swelling curve, ${k}_{3}$ the specific time at which the overshoot occurs.

Analysing the time at which the maximum is reached (last column in Table 6), the following considerations can be deduced:

- In acidic environment, highly cross-linked samples swell slowly. This trend agrees with the results of Diez-Peña et al. [52], where longer times to equilibrium conditions were also found for highly cross-linked hydrogels. This behaviour is most probably due to the stronger interactions that “protect” the hydrogen-bounded complexes from breakage. This can also be due to a higher stability of hydrogen bonds themselves that prevents water diffusion into the samples. As a matter of fact, the maximum swelling ratios of highly cross-linked hydrogels (1–3 and 1–4) are smaller than those of slightly cross-linked ones.
- At pH close to neutrality, a larger difference between samples 2–2 and 1–2 compared to that between 1–3 and 1–4 can be observed. This behaviour supports the existence of a critical value of crosslinking extent, even though additional experiments should be done to identify such threshold value.
- At neutral pH, swelling is controlled by the chemical crosslinking. Hydrogels 2–2 and 1–2 show larger swelling compared to samples 1–3 and 1–4, as confirmed by the peak times. The stiffer hydrogels retain less water, and the time needed to overcome the maximum is shorter. The opposite occurs for weaker hydrogels.
- In basic environment, the times at which the peak is reached are almost the same. In fact, under such conditions, the hydrogels are held together mainly through electrostatic interactions caused by deprotonation of the carboxylic groups. The ratio polyacrylic acid/crosslinker is different in the four samples; since no carboxylic groups are present in the crosslinker, the quantity of carboxyl groups is almost the same for every sample because the amount of carbomer is constant.

**Fig. 4:**
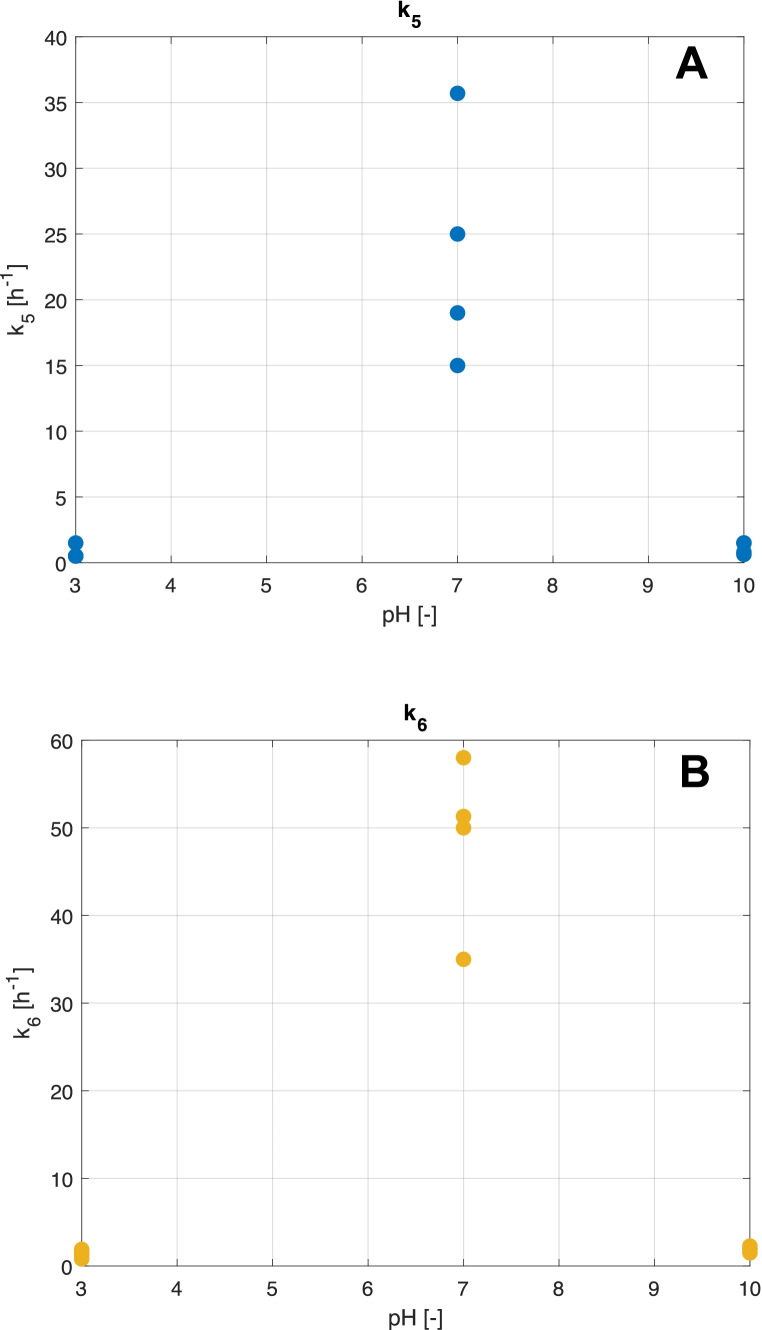
Effect of pH on the estimated values of the kinetic constants of swelling (*k*_5_) **A** and deswelling (*k*_6_) **B**

Let us now consider the role of the last two kinetic constants, ${k}_{5}$ and ${k}_{6}$. Their values determine the second portion of the swelling curve, where the final equilibrium is established. In very acidic or basic media, independently upon the chosen formulation, the hydrogel is quite stiff, and water absorption is extremely slow. On the other hand, at pH values close to neutrality, the structural properties are weaker, and the swelling/deswelling rates are larger. These behaviours are reflected by the estimated values of *k*_5_ and *k*_6_, as reported in Fig. 4. Furthermore, the ratio between the two kinetic constants remains almost equal at each value of pH and crosslinking density, meaning that, the ratio between the rates of release and recovery of water is similar independently from the hydrogel formulation. However, the external environment pH affects the nominal velocity of water release and uptake. At neutral pH, the structure is more flexible; hence, these kinetic step rates are larger compared to other analysed pH conditions where hydrogels present more rigid structures which hinder the release-uptake kinetic.

**Fig. 5:**
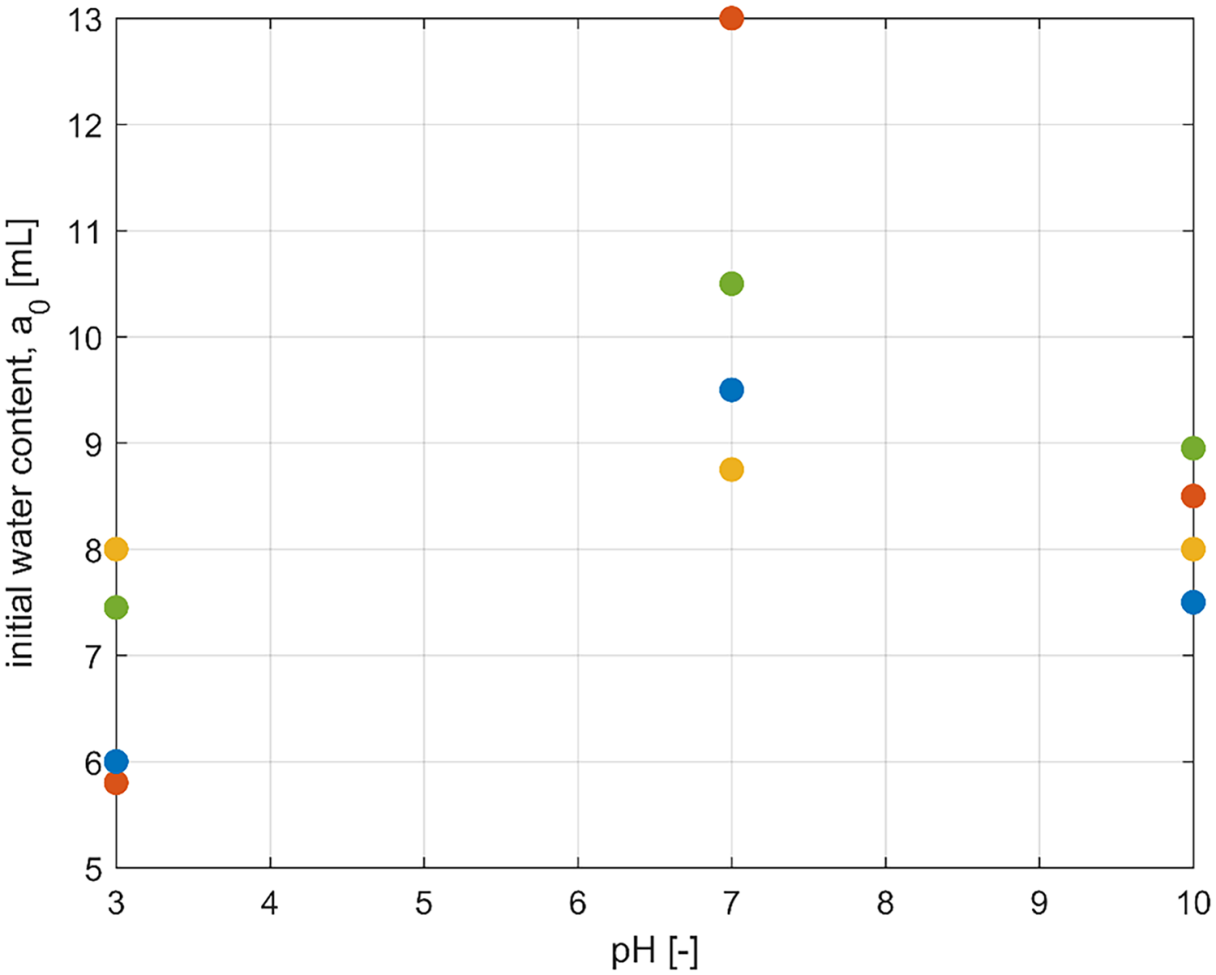
Effect of pH on the initial water content (*a*_0_) for all samples: hydrogel 2–2 (red), 1–2 (green), 1–3 (blue) and 1–4 (yellow)

Finally, some considerations on the structural properties of the hydrogels are helpful to describe the impact of the initial water content *a*_0_ on the model predictions. Water diffusion and crosslinking density are strongly dependent from each other, and several papers are available in the literature discussing water diffusion inside hydrogels [2]. In general, the diffusion coefficients inside the hydrogel drop at increasing crosslinking density, since the number of vacancies available for diffusion decreases. Accordingly, the hydrogels entrap less solvent at low and high pH values. On the contrary, at pH values close to neutrality, swelling is much more relevant, and the chemical crosslinking density controls the amount of water able to diffuse into the hydrogel at the beginning of the experiment: the more cross-linked the hydrogel, the lower its capability to absorb water. The estimated values of *a*_0_ shown in Fig. 5 reflect the previous arguments.

### Drug delivery

**Fig. 6:**
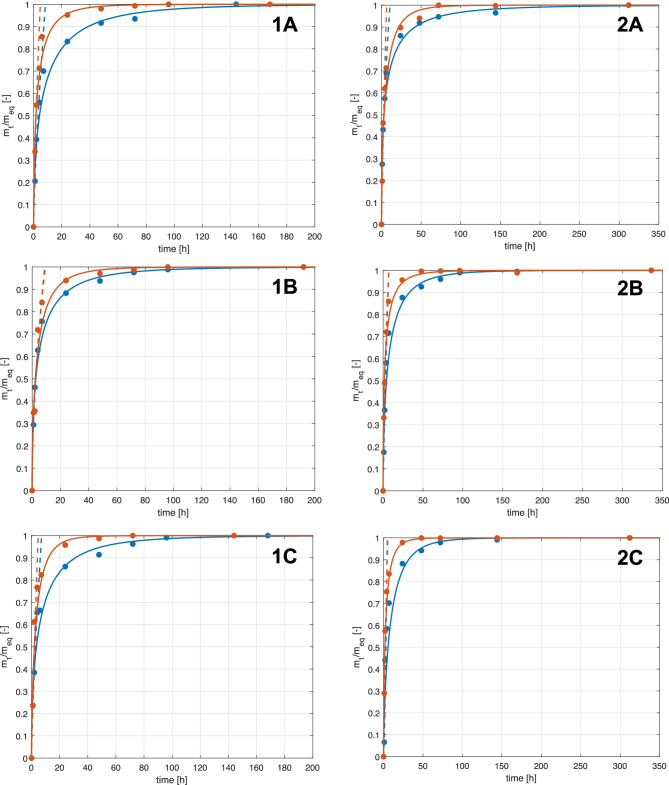
Comparison of RhB (1) and Esther-RhB (2) release for hydrogel 2–2 (blue) and 1–2 (red) at pH = 3 **A**, pH = 7 **B** and pH = 10 **C** using Weibull refitting function (solid lines) as in Kosmidis and coworkers [31, 46–48] and power-law (dashed lines) as in Peppas and coworkers [29, 44, 45]

Concerning drug delivery, the model based on the Weibull function previously mentioned has been compared with a classical power-law model proposed by Peppas and coworkers [29, 44]. Also in this case, the experimental values have been taken from Mauri et al. [31]. Two different rhodamine B (RhB) loadings have been taken into consideration: the first one obtained by loading the drug inside the hydrogel matrix by diffusion, while the second one exploits the formation of an ester bond, thus chemically linking the drug to the hydrogel matrix. Results showing the comparison between the different models (Weibull and power-law refit) are shown in Fig. 6.

As already mentioned, the power-law approach properly describes the behaviour of the drug release at the beginning, until $\frac{m\left(t\right)}{{m}_{eq}}$ ≤ 0.6 ÷ 0.7. Beyond this threshold value, the predictions of the power law model become unreliable, while those of the model based on the Weibull curve agree well with the complete experimental profile. Moreover, as shown in Fig. 6, all the Weibull functions fit carefully the experimental data close to the origin as well as to equilibrium conditions, while discrepancies appear in the central region of the plots. Such discrepancies may be due to the rearrangement of the inner hydrogel structure which affects the swelling behaviour and the operative diffusion regime.

Following the approach proposed by Papadopoulou et al. [48], a relation between the parameters *n* and *b* (estimated through the power law and the Weibull curve, respectively) exists. According to our results, the *n* values agree with the predictions based on Deborah number: the diffusion is governed by a non-Fickian regime together with other phenomena, such as the swelling of the polymeric matrix and the solvent penetration into the network. The main differences arise when considering the *b* parameters. As reported in [47], a linear relation between exponent *n* and *b* parameters has been found out. In Tables 7 and 8, the *b* values estimated using this linear interpolation are reported (column “regression”). Looking at the ratio between regressed and fitted values of the parameter *b*, it ranges from 1.41 to 3.33, with an average value equal to 1.99. Regressed values about twice the fitted ones can be explained considering that Kosmidis et al. applied their model using the Higuchi law, which assumes negligible swelling during the drug release process. This is not the case for carbomer hydrogels that exhibit pronounced swelling as shown in the swelling dynamic results discussion. Therefore, in case of swellable hydrogels, the relation between *n* and *b* proposed by Kosmidis et al. still applies but asks for some correction. Namely, the same linear interpolation can be proposed in the case under examination, but the coefficients are doubled:

**Table 7:** Relation between the fitting parameter b and n for RhB release

**Sample**	*𝐧* **(–)**	**Fitted** *𝐛* **(–)**	**Regressed** *𝐛* **(–)**	**Ratio**
**RhB pH = 3**				
2–2	0.720	0.539	1.124	2.06
1–2	0.694	0.571	1.087	1.90
**RhB pH = 7**				
2–2	0.547	0.514	0.879	1.75
1–2	0.523	0.601	0.846	1.41
1–3	0.793	0.658	1.226	1.86
**RhB pH = 10**				
2–2	0.735	0.549	1.145	2.09
1–2	0.847	0.679	1.302	1.92

**Table 8:** Relation between the fitting parameter b and n for Esther-RhB release

**Sample**	*𝐧* **(–)**	**Fitted** *𝐛* **(–)**	**Regressed** *𝐛* **(–)**	**Ratio**
**Ester-bonded RhB pH = 3**				
2–2	0.532	0.460	0.859	1.88
1–2	0.824	0.629	1.270	2.02
**Ester-bonded RhB pH = 7**				
2–2	0.864	0.590	1.326	2.25
1–2	0.560	0.529	0.898	1.70
**Ester-bonded RhB pH = 10**				
2–2	1.58	0.699	2.328	3.33

17 $$b=0.7038\cdot n+0.5500$$

## Conclusions

In this work, focusing on hydrogels made of carbomer and elastamine, swelling and drug release behaviours have been analysed.

In the first case, it has been demonstrated that these hydrogels are strongly pH sensitive. Their swelling dynamics have been well described through a semi-empirical, literature model involving five adjustable parameters. The same model is also effectively predicting the overshoot of swelling, a phenomenon rarely encountered working with hydrogels. The cause of the overshooting has been identified as the interplay between physical and chemical crosslinks, with the extent of the first ones strongly dependent upon the medium pH.

Such understanding is of pivotal importance when dealing with drug delivery in these devices, due to the combination between non-Fickian diffusion and relaxation of the polymer matrix. The Weibull curve has been found to be a fitting function effective to describe the experimental data. On the other hand, the estimated values of the model parameters do not agree with those reported in previous literature works. Even though the numerical values are about half of those expected, still, the selected fitting procedure remains successfully applicable to the highly swelling hydrogels examined in this work.

## Supplementary Information

Below is the link to the electronic supplementary material.

Supplementary file1 (DOCX 791 KB)
