# Supplementary material for: Mathematical modelling of cross-linked polyacrylic-based hydrogels: physical properties and drug delivery
Source: Drug Deliv Transl Res. 2022 Feb 12;12(8):1928–42. doi: 10.1007/s13346-022-01129-2 (PMC9242946; doi:10.1007/s13346-022-01129-2)
Supplement: Supplementary file 1 — Supplementary file1 (DOCX 791 KB) [file 13346_2022_1129_MOESM1_ESM.docx]

**SUPPORTING INFORMATIONs**

*Experimental data*

Experimental data on swelling properties have been collected weighting the freeze-dried gel and then weighting at certain time points after being putted in contact with a thermodynamically compatible solvent (namely water or PBS). Swelling tests have been performed at all the three tested pH (3, 7 and 10) and the results can be observed in the following Tables (S1-S3).

| **Hydrogel at pH = 3** | | | | | | |
| --- | --- | --- | --- | --- | --- | --- |
| **Time [h]** | **0** | **2** | **4** | **6** | **24** | **48** |
| **2_2 [g]** | 0.0629 | 0.3663 | 0.3502 | 0.3898 | 0.3097 | 0.3417 |
| **1_2 [g]** | 0.0567 | 0.3988 | 0.4288 | 0.3530 | 0.3092 | 0.3269 |
| **1_3 [g]** | 0.0791 | 0.4590 | 0.4207 | 0.4888 | 0.4702 | 0.4700 |
| **1_4 [g]** | 0.0928 | 0.5025 | 0.4564 | 0.5275 | 0.5157 | 0.5123 |

**Table S1** – Swelling data for hydrogel samples at pH = 3.

| **Hydrogel at pH = 10** | | | | | | |
| --- | --- | --- | --- | --- | --- | --- |
| **Time [h]** | **0** | **2** | **4** | **6** | **24** | **48** |
| **2_2 [g]** | 0.0667 | 0.4210 | 0.4103 | 0.4347 | 0.3970 | 0.3836 |
| **1_2 [g]** | 0.0543 | 0.4781 | 0.4841 | 0.4789 | 0.4367 | 0.4238 |
| **1_3 [g]** | 0.0814 | 0.5301 | 0.6134 | 0.5808 | 0.4754 | 0.4863 |
| **1_4 [g]** | 0.0908 | 0.6096 | 0.6439 | 0.5403 | 0.4787 | 0.4238 |

**Table S2** – Swelling data for hydrogel samples at pH = 10.

| **Hydrogel at pH = 7** | | | | | | |
| --- | --- | --- | --- | --- | --- | --- |
| **Time [h]** | **2_2 [g]** | **2_2 [g]** | **1_2 [g]** | **1_3 [g]** | **1_3 [g]** | **1_4 [g]** |
| **0** | 0.0504 | 0.0677 | 0.0529 | 0.0741 | 0.0586 | 0.0776 |
| **0.33** | 0.2449 | 0.5122 | 0.3043 | 0.3447 | 0.2656 | 0.3110 |
| **1** | 0.4481 | 0.6752 | 0.5175 | 0.5645 | 0.4064 | 0.5339 |
| **2** | 0.5106 | 0.7372 | 0.5466 | 0.6186 | 0.5048 | 0.6632 |
| **3** | - | 0.7384 | - | - | - | - |
| **4** | 0.5843 | - | 0.5851 | 0.6389 | 0.5878 | 0.7437 |
| **6** | - | - | 0.5556 | - | - | 0.7194 |
| **7** | 0.5602 | - | - | 0.5857 | 0.6092 | - |
| **8.5** | - | 0.8285 | - | - | - | - |
| **24** | 0.5537 | 0.8129 | 0.5181 | 0.5440 | 0.5886 | 0.6138 |
| **48** | 0.4933 | 0.7110 | 0.4983 | 0.5056 | 0.5168 | 0.5658 |
| **72** | - | 0.7275 | - | - | - | 0.5359 |
| **120** | 0.5191 | - | 0.4561 | 0.5356 | - | - |
| **144** | - | - | 0.4817 | - | - | - |
| **192** | 0.4734 | - | 0.4608 | 0.4805 | 0.4921 | - |
| **216** | - | 0.7131 | - | - | - | 0.4839 |
| **336** | 0.4937 | - | 0.4415 | 0.4598 | 0.4946 | - |
| **360** | - | 0.7203 | - | - | - | 0.5070 |

**Table S3** – Swelling data for hydrogel samples at pH = 7.

Following the swelling tests, also release essays have been performed experimentally. Both physically entrapped chemically bonded RhB have been tested and results are shown in the following Tables.

| **RhB release** | | | | | | | |
| --- | --- | --- | --- | --- | --- | --- | --- |
|  | **pH = 3** | | **pH = 7** | | | **pH = 10** | |
| **Time [h]** | **2_2** | **1_2** | **2_2** | **1_2** | **1_3** | **2_2** | **1_2** |
| **0** | 0 | 0 | 0 | 0 | 0 | 0 | 0 |
| **1** | 0.0297 | 0.0443 | 0.0287 | 0.0399 | 0.0204 | 0.0326 | 0.0235 |
| **2** | 0.0567 | 0.0717 | 0.0451 | 0.0407 | 0.0365 | 0.0843 | 0.0383 |
| **4** | 0.0806 | 0.0933 | 0.0613 | 0.0825 | 0.0611 | 0.1056 | 0.0652 |
| **6** | 0.1011 | 0.1119 | 0.0739 | 0.0965 | 0.0784 | 0.1137 | 0.0663 |
| **24** | 0.1203 | 0.1247 | 0.0863 | 0.1078 | 0.0902 | 0.1317 | 0.0859 |
| **48** | 0.1323 | 0.1283 | 0.0916 | 0.1113 | 0.0931 | 0.1358 | 0.0913 |
| **72** | 001350 | 0.1300 | 0.0953 | 0.1132 | 0.0941 | 0.1376 | 0.0961 |
| **96** | - | 0.1309 | 0.0967 | 0.1147 | 0.0954 | - | 0.0990 |
| **144** | 0.1445 | - | - | - | - | 0.1376 | - |
| **168** | - | 0.1310 | - | - | - | - | 0.0998 |
| **192** | - | - | 0.1179 | 0.1147 | 0.0955 | - | - |

**Table S4** – RhB release experimental data.

| **Ester bonded RhB release** | | | | | | |
| --- | --- | --- | --- | --- | --- | --- |
|  | **pH = 3** | | **pH = 7** | | **pH = 10** | |
| **Time [h]** | **2_2** | **1_2** | **2_2** | **1_2** | **2_2** | **1_2** |
| **0** | 0 | 0 | 0 | 0 | 0 | 0 |
| **1** | 0.0488 | 0.0218 | 0.0241 | 0.0416 | 0.0085 | 0.0323 |
| **2** | 0.0768 | 0.0511 | 0.0503 | 0.0612 | 0.0571 | 0.0641 |
| **4** | 0.1022 | 0.0685 | 0.0797 | 0.0903 | 0.0758 | 0.0840 |
| **6** | 0.1230 | 0.0788 | 0.0984 | 0.1078 | 0.0910 | 0.0932 |
| **24** | 0.1531 | 0.0993 | 0.1205 | 0.1198 | 0.1143 | 0.1091 |
| **48** | 0.1634 | 0.1040 | 0.1272 | 0.1249 | 0.1221 | 0.1115 |
| **72** | 0.1685 | 0.1105 | 0.1319 | 0.1250 | 0.1267 | 0.1114 |
| **96** | - | - | 0.1360 | 0.1252 | - | - |
| **144** | 0.1717 | 0.1105 | - | - | 0.1284 | 0.1115 |
| **168** | - | - | 0.1367 | 0.1250 | - | - |
| **312** | 0.1780 | 0.1104 | - | - | 0.1296 | 0.1114 |
| **336** | - | - | 0.1374 | 0.1250 | - | - |

**Table S5** – chemically bonded RhB release data.

*Hydrogel structural properties*

Using the Flory-Rehner theory, some structural properties of the hydrogels have been computed, checking their accordance with the experimental data. One of the most important features of such system is the mesh size $\xi$:

**Figure S1** – Effect of the solution pH on the mesh size. Each color is representative of a specific hydrogel formulation: red is 2_2, green is 1_2, blue is 1_3 and yellow is 1_4 respectively. The solid lines represent the model prediction. The mesh is narrow for high and low pH values, while at pH close to neutrality the mesh size increases sensibly.

From Figure S1 it can be observed how the solution pH strongly influences the final value of the mesh size. Furthermore, also the formulation plays an important role: increasing the crosslinking density, the hydrogel exhibits a lower swelling capacity, thus resulting in a more compact structure (lower mesh size).

The molecular weight between crosslinks is another parameter that should be important to analyze. Its trend is similar to that of the mesh size, as it can be observed in Figure S2.

**Figure S2** – Effect of the solution pH on the molecular weight between crosslinks. Each color is representative of a specific hydrogel formulation: red is 2_2, green is 1_2, blue is 1_3 and yellow is 1_4 respectively. The solid lines represent the model prediction. The larger the molecular weight between crosslinks, the lower the crosslinking densities.

If the hydrogel is highly crosslinked, then the value of the molecular weight between crosslinks decreases since the connections between different chains become more frequent. On the contrary, as discussed before these hydrogels exhibit a swelling behaviour that is affected by the solution pH. Another quantity representative of the swelling behaviour is the polymer volume fraction, and its trend is reported in Figure S3.

**Figure S3** – Effect of the solution pH on the polymer volume fraction. Each color is representative of a specific hydrogel formulation: red is 2_2, green is 1_2, blue is 1_3 and yellow is 1_4 respectively. The solid lines represent the model prediction. It can be observed how the hydrogel exhibits an intrinsic lower capacity of water storage at pH values far from the equivalence one.

*Swelling dynamics*

**Figure S4** – Swelling curves of hydrogel 2_2, experimental data fitting.

The equation governing the swelling dynamics is Eqn. (11), function of five adjustable parameters. Those parameters have been evaluated for all the tested formulations and at the 3 different pH values (3, 7 and 10). Using the fitting parameters reported in the main text, the model has been used for evaluating the evolution of swelling ratio during time, for all the four tested formulations at the three pH values.

**Figure S5** – Swelling curves of hydrogel 1_2, experimental data fitting.

**Figure S6** – Swelling curves of hydrogel 1_3, experimental data fitting.

**Figure S7** – Swelling curves of hydrogel 1_4, experimental data fitting.

In Figures S4-S7 all the comparisons with experimental data have been reported.

As reported in the main text, four different types of water are present in this system. Taking as an example hydrogel 1_2, their evolution during time has been evaluated. Results are shown in Figure S8 at the three different pH values.

**Figure S8** – Time evolution of the different types of water in hydrogel 1_2 at various pH values.

All the trends predicted by the model can be explained. The first type of water, $A_{1}$ starts from the initial value equal to $a_{0}$ and monotonically decreases. This is due to the hydrogel swelling and water uptaking, thus increasing the second type of water content $A_{2}$. The volume increase of the solid network is so large that the elastic contribution starts to reduce the swelling and induce the hydrogel shrinking. Therefore, the secondary structure, that is composed of the third type of water $A_{3}$ starts to become dominant after the swelling peak. At this point, a fraction of water, i.e. $A_{4}$, is released by the system up to achievement of equilibrium conditions.

*Drug delivery*

Two different models have been tested and compared in this work: power law and the Weibull curve. For applying a linear regression on the experimental data, the two models need to be linearized. The independent variable is time, while the dependent one is the ratio $\frac{m(t)}{m_{eq}}$, defined in the following as $r$. The linearized form of the power law model is the following:

$$\ln r= \ln k+n\ln t$$

While for the Weibull curve the expression becomes:

$$\ln\left[ -\ln(1-r) \right]= \ln a+b\ln t$$

In the origin of the axes both models exhibit a singularity point, since $r=0$ at $t=0$. Furthermore, the power law model shows a good accordance with the experimental points only when $r<0.60$, thus only a small fraction of experimental points is useful for this interpolation. Fitting results can be observed in the following Tables, along with the different $R^{2}$ values for the various fittings.

| **Rhodamine – pH = 3** | | | |
| --- | --- | --- | --- |
| **Hydrogel 2_2** | | **Hydrogel 1_2** | |
| **a** | 0.3206 | **a** | 0.5153 |
| **b** | 0.5388 | **b** | 0.5712 |
| **n** | 0.7204 | **n** | 0.6944 |
| **k** | 0.2160 | **k** | 0.3383 |
| $\boldsymbol{R}^{\boldsymbol{2}}$ **Weibull l.** | 0.9480 | $\boldsymbol{R}^{\boldsymbol{2}}$ **Weibull l.** | 0.9635 |
| $\boldsymbol{R}^{\boldsymbol{2}}$ **Weibull** | 0.9960 | $\boldsymbol{R}^{\boldsymbol{2}}$ **Weibull** | 0.9980 |
| $\boldsymbol{R}^{\boldsymbol{2}}$ **power l.** | 0.9720 | $\boldsymbol{R}^{\boldsymbol{2}}$ **power l.** | 1.000 |

**Table S6** – Fitting parameters for RhB release at pH = 3. Weibull l. is the interpolation using the linearized model, while power l. is the power law model.

| **Rhodamine – pH = 7** | | | | | |
| --- | --- | --- | --- | --- | --- |
| **Hydrogel 2_2** | | **Hydrogel 1_2** | | **Hydrogel 1_3** | |
| **a** | 0.4251 | **a** | 0.4224 | **a** | 0.3274 |
| **b** | 0.5138 | **b** | 0.6010 | **b** | 0.6578 |
| **n** | 0.5469 | **n** | 0.5228 | **n** | 0.7928 |
| **k** | 0.3011 | **k** | 0.3106 | **k** | 0.2158 |
| $\boldsymbol{R}^{\boldsymbol{2}}$ **Weibull l.** | 0.9800 | $\boldsymbol{R}^{\boldsymbol{2}}$ **Weibull l.** | 0.9404 | $\boldsymbol{R}^{\boldsymbol{2}}$ **Weibull l.** | 0.9587 |
| $\boldsymbol{R}^{\boldsymbol{2}}$ **Weibull** | 0.9981 | $\boldsymbol{R}^{\boldsymbol{2}}$ **Weibull** | 0.9958 | $\boldsymbol{R}^{\boldsymbol{2}}$ **Weibull** | 0.9969 |
| $\boldsymbol{R}^{\boldsymbol{2}}$ **power l.** | 0.9882 | $\boldsymbol{R}^{\boldsymbol{2}}$ **power l.** | 0.9578 | $\boldsymbol{R}^{\boldsymbol{2}}$ **power l.** | 0.9988 |

**Table S7** – Fitting parameters for RhB release at pH = 7. Weibull l. is the interpolation using the linearized model, while power l. is the power law model.

| **Rhodamine – pH = 10** | | | |
| --- | --- | --- | --- |
| **Hydrogel 2_2** | | **Hydrogel 1_2** | |
| **a** | 0.3487 | **a** | 0.4252 |
| **b** | 0.5491 | **b** | 0.6792 |
| **n** | 0.7353 | **n** | 0.8470 |
| **k** | 0.2342 | **k** | 0.2676 |
| $\boldsymbol{R}^{\boldsymbol{2}}$ **Weibull l.** | 0.9593 | $\boldsymbol{R}^{\boldsymbol{2}}$ **Weibull l.** | 0.9285 |
| $\boldsymbol{R}^{\boldsymbol{2}}$ **Weibull** | 0.9963 | $\boldsymbol{R}^{\boldsymbol{2}}$ **Weibull** | 0.9952 |
| $\boldsymbol{R}^{\boldsymbol{2}}$ **power l.** | 0.9994 | $\boldsymbol{R}^{\boldsymbol{2}}$ **power l.** | 0.9072 |

**Table S8** – Fitting parameters for RhB release at pH = 10. Weibull l. is the interpolation using the linearized model, while power l. is the power law model.

These three Tables include the results obtained with the RhB freely entrapped inside the hydrogel matrix. The same evaluations have been performed also for the chemically linked RhB through an ester bond. The results are shown in the following Tables.

| **Ester-Rhodamine – pH = 3** | | | |
| --- | --- | --- | --- |
| **Hydrogel 2_2** | | **Hydrogel 1_2** | |
| **a** | 0.4118 | **a** | 0.3308 |
| **b** | 0.4596 | **b** | 0.6290 |
| **n** | 0.5322 | **n** | 0.8243 |
| **k** | 0.2822 | **k** | 0.2167 |
| $\boldsymbol{R}^{\boldsymbol{2}}$ **Weibull l.** | 0.9662 | $\boldsymbol{R}^{\boldsymbol{2}}$ **Weibull l.** | 0.9551 |
| $\boldsymbol{R}^{\boldsymbol{2}}$ **Weibull** | 0.9974 | $\boldsymbol{R}^{\boldsymbol{2}}$ **Weibull** | 0.9976 |
| $\boldsymbol{R}^{\boldsymbol{2}}$ **power l.** | 0.9830 | $\boldsymbol{R}^{\boldsymbol{2}}$ **power l.** | 0.9260 |

**Table S9** – Fitting parameters for chemically linked RhB release at pH = 3. Weibull l. is the interpolation using the linearized model, while power l. is the power law model.

| **Ester-Rhodamine – pH = 7** | | | |
| --- | --- | --- | --- |
| **Hydrogel 2_2** | | **Hydrogel 1_2** | |
| **a** | 0.2923 | **a** | 0.5335 |
| **b** | 0.5898 | **b** | 0.5292 |
| **n** | 0.8642 | **n** | 0.5600 |
| **k** | 0.1833 | **k** | 0.3314 |
| $\boldsymbol{R}^{\boldsymbol{2}}$ **Weibull l.** | 0.9590 | $\boldsymbol{R}^{\boldsymbol{2}}$ **Weibull l.** | 0.9300 |
| $\boldsymbol{R}^{\boldsymbol{2}}$ **Weibull** | 0.9970 | $\boldsymbol{R}^{\boldsymbol{2}}$ **Weibull** | 0.9977 |
| $\boldsymbol{R}^{\boldsymbol{2}}$ **power l.** | 0.9827 | $\boldsymbol{R}^{\boldsymbol{2}}$ **power l.** | 0.9999 |

**Table S10** – Fitting parameters for chemically linked RhB release at pH = 7. Weibull l. is the interpolation using the linearized model, while power l. is the power law model.

| **Ester-Rhodamine – pH = 10** | | | |
| --- | --- | --- | --- |
| **Hydrogel 2_2** | | **Hydrogel 1_2** | |
| **a** | 0.2049 | **a** | 0.4906 |
| **b** | 0.6993 | **b** | 0.6324 |
| **n** | 1.5760 | **n** | 0.6900 |
| **k** | 0.0862 | **k** | 0.3103 |
| $\boldsymbol{R}^{\boldsymbol{2}}$ **Weibull l.** | 0.8450 | $\boldsymbol{R}^{\boldsymbol{2}}$ **Weibull l.** | 0.9524 |
| $\boldsymbol{R}^{\boldsymbol{2}}$ **Weibull** | 0.9930 | $\boldsymbol{R}^{\boldsymbol{2}}$ **Weibull** | 0.9985 |
| $\boldsymbol{R}^{\boldsymbol{2}}$ **power l.** | 0.9451 | $\boldsymbol{R}^{\boldsymbol{2}}$ **power l.** | 0.9410 |

**Table S11** – Fitting parameters for chemically linked RhB release at pH = 10. Weibull l. is the interpolation using the linearized model, while power l. is the power law model.
